# The Effect of Octreotide on Urine Output During Orthotopic Liver Transplantation and Early Postoperative Renal Function; A Randomized, Double-Blind, Placebo-Controlled Trial

**DOI:** 10.5812/hepatmon.12787

**Published:** 2013-09-18

**Authors:** Mohammad Ali Sahmeddini, Afshin Amini, Nima Naderi

**Affiliations:** 1 Shiraz Anesthesiology and Intensive Care Research Center, Shiraz University of Medical Sciences, Shiraz, IR Iran

**Keywords:** Liver Transplantation, Octreotide, Acute Kidney Injury

## Abstract

**Background:**

Maintenance of the adequate intraoperative renal perfusion is very important during Orthotopic Liver Transplantation (OLT) to prevent acute renal failure.

**Objectives:**

For the first time, this study was designed to survey the effects of octreotide on urine output during anesthesia for OLT and early postoperative renal function.

**Patients and Methods:**

In this randomized double-blind placebo controlled clinical trial, 79 of 89 patients who underwent OLT and fulfilled the study requirement were randomly allocated into two groups. In the octreotide group, the patients received octreotide infusion from the start of the operation. On the other hand, the control group patients received physiologic saline infusion instead of octreotide. The Mean Arterial Pressure (MAP), heart rate, urine output, norepinephrine usage, and dosage during the three stages of OLT, and baseline and postoperative creatinine were recorded and compared between the two groups.

**Results:**

No significant differences were found between the two groups regarding the demographic characteristics and graft factors (P > 0.05). However, urine output and MAP during the three stages of OLT were significantly higher in the octreotide group compared to the control group (P < 0.05). Moreover, no significant difference was observed between the two groups regarding baseline as well as postoperative creatinine (P > 0.05).

**Conclusions:**

The results demonstrated that octreotide infusion during anesthesia for OLT not only augmented the vasoconstriction effect of norepinephrine to increase MAP, but also maintained better renal perfusion and urine output during the operation.

## 1. Background

Acute renal insufficiency is one of the most serious complications following Orthotopic Liver Transplantation (OLT) (1-3). The incidence of acute renal insufficiency after OLT varies from 25% to 70% in different centers due to using different diagnostic methods (4, 5). Previous studies showed that the mortality rate following OLT was two times more in patients who developed ARF compared to those without ARF (5, 6). ARF has been shown to be more common among the OLT patients with a higher preoperative serum creatinine (Cr) level, greater need for blood transfusions during the operation, and more episodes of hypotension during the operation (5-7). Anesthesiologists have to be responsible for maintaining stable hemodynamic status intraoperatively to preserve renal perfusion and urine output (8).

One of the important causes of renal hypo-perfusion is splanchnic vasodilation with subsequent intrarenal vasoconstriction which is usually detected in patients with cirrhosis and portal hypertension (9, 10). Therefore, to maintain renal perfusion, we should increase splanchnic vascular tone with vasoconstrictor (11-13). Some studies have suggested vasopressin as a splanchnic vasoconstrictor, but severe ischemic complications have made vasopressin unfavorable (13, 14). The acute administration of vasopressin analogues, such as terlipress or ornipressin, is still controversial (15, 16). On the other hand, in patients with cirrhosis, lack of response to vasoconstrictors, such as norepinephrine, in the splanchnic area is due to the increased level of both endothelial (nitric oxide) and nonendothelial vasodilators (glucagon) (15).

Octreotide is an inhibitor of the releasing vasodilator peptides, such as glucagon and vasoactive intestinal peptide (15). Some animal studies have shown that octreotide decreases the level of glucagon, eventually improving the vasoconstrictors effect of norepinephrine in patients with cirrhosis (17). However, its use during general anesthesia for OLT has not been reported in the literature.

## 2. Objectives

This randomized double blind clinical trial was designed to evaluate the effect of combination of octreotide and norepinephrine on urine output and early postoperative renal function in the patients undergoing deceased OLT.

## 3. Materials and Methods

### 3.1. Ethical Considerations

This study was approved by our institutional ethics committee. The purpose of the study was explained to the patients and their families, and written informed consents were obtained.

### 3.2. Subjects

The eligible patients were all adults aging 16 years and above. They were candidates for orthotopic deceased donor liver transplantation from September 2011 to April 2012 in Shiraz Organ Transplantation Center. The exclusion criteria of the study were ischemic heart disease, systemic hypertension, renal failure, heart block, diabetes mellitus, and surgical techniques other than piggyback.

### 3.3. Study Design

This single center, double-blind, placebo-controlled, parallel- groups clinical trial with balanced randomization (IRCT 2012120411662N1) was conducted in Iran. The patients were randomly assigned to two parallel groups to receive either norepinephrine alone or a combination of norepinephrine and octreotide.

### 3.4. Outcome Measures

The primary outcome with respect to the efficacy of octreotide in maintaining renal blood flow perfusion was the urine output measurement during the three stages of the operation. Yet, the secondary outcome was serum creatinine measured by jaffe/kinetic method on the 1st and 3rd days post operation.

### 3.5. Sample Size Calculation

We did not find any trials regarding the use of octreotide during anesthesia for liver transplantation in the review of the literature to calculate the sample size therefore, we recruited all the eligible patients from September 2011 to April 2012. Among eighty nine patients with end-stage liver disease candidates for orthotropic deceased donor liver transplantation during this period, just seventy nine patients fulfilled the study requirements.

### 3.6. Randomization

The patients were randomly assigned to the two study groups through simple randomization using computerized random numbers. Each of the patients with a 1:1 ratio was allocated to the octreotide or the control group. A nurse anesthetist who was not involved in data collection and treatment performed the patients' enrollment and assignment into the treatment groups.

### 3.7. Intervention

Anesthesia was induced with thiopental (5mg/Kg), fentanyl (2μg/Kg), and midazolam (0.03mg/Kg), and pancuronium (0.1 mg/Kg) was used for neuromuscular blockade. In addition, ventilation was maintained by 50% air-50% oxygen mixture plus isoflurane. Cardiovascular function was monitored by electrocardiogram, radial artery catheter, and the Central Venous Pressure (CVP) via the right internal jugular vein through the double lumen central venous access line. Besides, hepatectomy was performed for all the patients by using the piggy-back technique.

The octreotide and placebo (normal saline) were in 50 ml syringes which were identical in appearance. They were prepared by a nurse anesthetist who was not participating in the study. The 50 ml syringes with A label contained 50 microgram octreotide diluted with normal saline in the total volume of 50 ml, while the 50 ml syringes with B label contained just 50 ml normal saline. The patients and the research assessor were not aware of the contents of the syringes A and B.

In the octreotide group, after induction of anesthesia, octreotide was started with 50 microgram dosage in 50 ml syringes intravenously (IV) in 15 minutes followed by 50 microgram per hour in 50 ml syringes with A label. In the control group, on the other hand, normal saline in 50 ml syringes with B label was started. In both groups, 5% albumin and fresh frozen plasma were administrated to maintain the CVP at ≥ 10 cmH2O. In case the Mean Blood Pressure (MAP) dropped to less than 60 mmHg, the patients were given norepinephrine as a vasoconstrictor with an initial dose of 0.05 μg /kg/min. The dosage was increased until the MAP was maintained at more than 60 mmHg.

### 3.8. Study Measurements

Hepatectomy phase was defined from the beginning of the operation up to clamping the inferior vena cava and portal vein. In addition, an-hepatic phase started from clamping the inferior vena cava and portal vein and liver removal up to the declamping of the inferior vena cava and portal vein. Finally, neo-hepatic phase began by declamping of the inferior vena cava and portal vein and reperfusion of the new liver up to the end of the operation.

The cold ischemic time was defined as the period from aortic cross-clamping and perfusion with the preservation solutions in the donor up to the time the liver was taken out from cold preservative fluid. From this time, warm ischemic time was started and continued up to the completion of the anastomosis and portal reperfusion. Thus, the total ischemic time was calculated as the period from the aortic cross-clamping and perfusion with the preservative solutions in the donor up to the completion of the anastomosis and portal reperfusion.

The hemodynamic parameters; i.e. MAP, heart rate, central venous pressure of the patients, and dosage of norepinephrine, which were used to maintain MAP ≥ 60 mmHg were recorded during the three stages of transplantation. Furthermore, the patients' urine output (ml/kg/hr) was recorded at the end of each stage of the liver transplantation and their serum creatinine was recorded on the first and 3rd days of the operation. Also, the patients were followed for using Chronic Renal Replacement Therapy (CRRT) until 5 days post operation.

### 3.9. Statistical Analysis

The analyses were performed by using the SPSS statistical software, 18.0 (Statistic package for Mac OS X version). Repeated measures analysis of variance was used for intergroup comparison of hemodynamic parameters. In addition, unpaired t-test or Mann-Whitney U test was used for continuous variables, and χ^2^ test was used for categorical ones. All values were presented as means ± SD or median (interquartile), and P < 0.05 was considered as statistically significant.

## 4. Results

### 4.1. The Enrolled Patients

**Figure 1.**
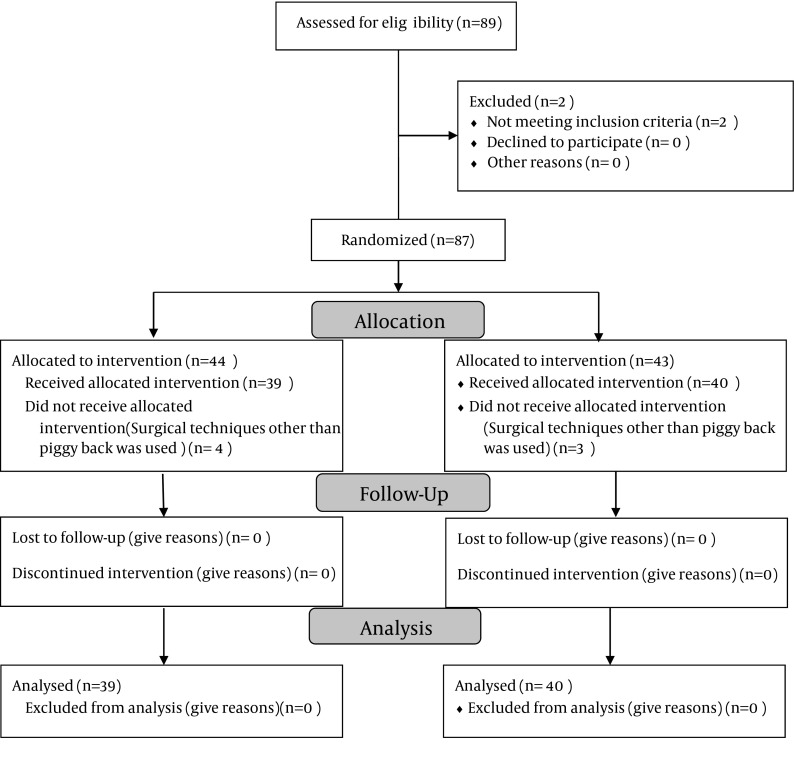
Flowchart of the Patients According to the Consort Guidelines

Among 89 patients who underwent OLT from September 2011 to April 2012, seventy nine ones fulfilled the study requirement. They were randomly allocated into control (n = 39) and octreotide groups (n = 40). Exclusion criteria were diabetes mellitus (n = 2) and surgical technique other than piggy back (n = 8) (Figure 1).

### 4.2. Medication Adverse Effects and Complications

An independent senior attending reviewed the unblinded data for patient safety. Also, he followed the patients in the octreotide group during the trial for recording the possible octreotide complications. However, no changes occurred in the study method after the trial commencement.

### 4.3. Study Findings

The demographic variables including sex, age, Meld score, baseline creatinine, and volume of ascites fluid are summarized in Table 1. No significant differences were found between the two groups regarding the demographic variables (P > 0.05). Besides, no significant difference was observed between the control and octreotide groups regarding the graft factors (P > 0.05) (Table 2). The two groups were also similar concerning the duration of the operation, cold and warm ischemic times, and total ischemic time (P > 0.05) (Table 2).

**Table 1.** Demographics Variables of the Recipients of Octreotide and the Control Groups ^a ^

Variables	Control Group (N = 39)	Octreotide Group (N = 40)	P value
**Sex (Male/Female)**	26/13	23/17	0.41
**Age, y**	38.18 ± 12.46	41.75 ± 17.14	0.33
**MELD Score**	21.66 ± 5.12	20.60 ± 7.08	0.48
**Ascites (ml)**	0 - 12000	0-8500	0.15
**Baseline Creatinine**	0.43 ± 0.67	0.56 ± 0.55	0.23

^a^ All the data are presented as mean ± standard deviation, Median (interquartile range)

**Table 2.** Demographic Characteristics of the Deceased Donors of Both Groups, and Operation Times ^a ^

	Control group (N = 39)	Octreotide group (N = 40)	95%CI of difference	P value
**Donor Age, y**	28.56 ± 7.32	30.12 ± 6.85	-2.87 to 9.56	0.38
**Fatty change of liver, %**	4.50 ± 1.8	5.10 ± 0.6	-0.55 to 2.32	0.61
**Graft weigh/recipient weight**	1.15 ± 0.18	1.20 ± 0.25	-0.01 to 1.05	0.43
**Warm ischemic time, min**	45.24 ± 9.05	43.42 ± 9.98	-5.31 to 8.54	0.44
**Total ischemic time, H**	9.5 ± 1.8	10.8 ± 1.4	-1.56 to 2.78	0.29
**Cold ischemic time, min**	375 (120 - 735)	400 (120 - 690)		0.26

^a^ All the data are reported as mean ± standard deviation or median (interquartile range)

Moreover, the results revealed no statistically significant differences between the two groups regarding estimated blood loss, the volume of transfused crystalloid and albumin during the operation, and the volume of blood product transfusion (packed red blood cell and fresh frozen plasma) (P > 0.05) (Table 3).

**Table 3.** Fluid Therapy, Blood and Blood Components Therapy in Both Groups ^a ^

	Control group (n = 39)	Octreotide group (n = 40)	P value
**Estimated Blood loss, ml**	2800 (1500-6500)	3000 (1600-7000)	0.67
**Crystalloids, ml**	3600 (3000 - 4500)	3400 (3100 - 5000)	0.56
**Albumin 5%, gram**	70 (60 - 90)	70 (60 - 90)	0.45
**PRBC ^b^, ml**	2500 (1350 - 3220)	2850 (1500-6700)	0.38
**FFP ^b^, ml**	1600 (1000 - 2000)	1400 (1000-1800)	0.74

^a^ All the data are reported as median (interquartile range)

^b^Abbreviations: PRBC, Pack red blood cell; FFP, Fresh ferozen plasma

The study findings indicated that MAP was significantly higher in the octreotide group compared to the control group (P < 0.05) (Table 4). However, all the patients in the control and octreotide groups received norepinephrine with dosages of 0.23 ± 0.09 and 0.25 ± 0.06 (μg/kg/min), respectively, and no statistically significant difference was observed between the two groups regarding norepinephrine infusion dosage (P = 0.4).

**Table 4.** Hemodynamic Parameters During the Three Stages of Liver Transplantation in Both Groups ^a ^

Variables	Control group (n = 39)	Octreotide group (n = 40)	95% CI ^b^ of difference	P value
**Hepatectomy phase**				
MAP ^b^, mmHg	78.48 ±11.69	86.09 ± 15.20	7.12 to 8.01	0.03
Hear rate, Beats/Min	93.93 ± 12.85	90.90 ± 18.81	-8.76 to 11.2	0.78
CVP ^b^ , mmHg	10.14 ± 1.23	9.89 ± 1.09	-2.45 to 10.89	0.59
**An-hepatic phase**				
MAP, mmHg	70.39 ± 8.70	76.24 ± 11.76	7.05 to 9.81	0.04
Hear rate, Beats/Min	95.75 ± 12.56	94.93 ± 15.42	-13.23 to 10.5	0.69
CVP, mmHg	8.85 ± 1.76	9.01± 2.1	-1.99 to 3.30	0.49
**Neo-hepatic phase**				
MAP, mmHg	78.45 ± 9.38	80.51 ± 8.27	5.11 to 2.70	0.04
Hear rate, Beats/Min	86.66 ± 11.77	88.42 ± 13.68	-10.55 to 9.50	0.51
CVP, mmHg	10.01 ± 1.01	11.01 ± 0.58	-2.17 to 4.45	0.67

^a^ All the values are presented as mean ± standard deviation

^b^ Abbreviations: MAP, Mean Arterial Pressure (mmHg); CVP, Central Venous Pressure (mmHg); CI, Confidence Interval

In the octreotide group, urine output (ml/kg/hr) was significantly higher compared to the control group during the three stages of the OLT and in the postoperative period (P < 0.05) (Table 5). However, no significant differences were found between the baseline and postoperative serum creatinine on the 1st and 3rd days of the operation in the two groups (P > 0.05) (Tables 1 and 5). Moreover, no patients in the two groups developed primary non function graft or required CRRT (P > 0.05).

**Table 5.** Urine Output During the Three Stages of Liver Transplantation and Postoperative Serum Creatinine ^a^

Variables	Control Group (n = 39)	Octreotide Group (n = 40)	95% CI of Difference	P value
**Urine Output in hepatectomy phase, ml/Kg/hr**	0.51 ± 0.25	0.99 ± 0.31	0.01 to 0.91	0.01
**Urine output in anhepatic phase, ml/Kg/hr**	0.25 ± 0.11	0.48 ± 0.19	0.02 to 0.78	0.04
**Urine output in neohepatic phase, ml/Kg/hr**	1.12 ± 0.67	1.78 ± 0.87	0.03 to 0.98	0.02
**Serum creatinine in the 1st day postoperation**	0.78 ± 0.38	0.75 ± 0.34	-0.15 to 0.31	0.65
**Serum creatinine in the 3rd day postoperation**	1.10 ± 0.25	1.08 ± 0.45	-0.5 to 0.67	0.45

^a^ All the values are reported as mean ± standard deviation.

## 5. Discussion

The results of this study revealed two interesting points in management of anesthesia for OLT. First, the combination of octreotide and norepinephrine has a major contribution to preserving renal perfusion and urine output during the operation. Second, this combination leads to maintenance of better MAP during anesthesia. The rationale for using octreotide in this study depends on the hypothesis that splanchnic vasodilatation in patients with cirrhosis is the primary event leading to systemic hypovolemia, and renal artery vasoconstriction subsequently decreasing the glomerular filtration rate (18). Due to the fact that urine output is the main monitoring of renal perfusion during anesthesia, it was used as a marker of renal perfusion during anesthesia for OLT in this study. However, postoperative serum creatinine was used as a marker of postoperative renal function.

Anesthesiologists usually use vasopressin or its analogues as a splanchnic vasoconstrictor to maintain renal function during the perioperative period of OLT. However, a recent animal study showed that vasopressin might cause ischemic necrosis with the infusion dose ≥ 0.04 U/min; therefore, vasopressin is not a safe drug. Moreover, vasopressin analogues, which are safer than vasopressin, are not available in many countries (17).

Nonetheless, two uncontrolled studies showed that octreotide in combination with α agonist agent midodrine or alone was an effective splanchnic vasoconstrictor to restore the renal function in patients with cirrhosis (15, 19). Of course, just five patients were enrolled into each study and both studies were nonrandomized. On the other hand, our study was randomized and double-blind, and was performed on a larger sample size, which are considered as the positive points of this study.

Kiser et al. showed that octreotide monotherapy was not effective in renal hemodynamic maintenance and restoration of renal function (20). Also, Pomier-Layrargues et al. showed that using octreotide alone did not have any effects on splanchnic vasculature and renal function (21). It is obvious that octreotide monotherapy does not affect splanchnic vasculature and renal function because octreotide is not a direct vasoconstrictor. As a matter of fact, octreotide has vasodilatation-inhibitory effects by inhibiting the release of glucagon. Therefore, octreotide was used in this study to augment the effect of norepinephrine as an α agonist agent.

Pomier-Layrargues et al. in their study demonstrated that octreotide needed at least 48 hours to start its effect on the renal function parameters, and the best result could be observed after 4-8 days (21). However, the hemodynamic effects of this drug on splanchnic and renal circulation are usually induced in a shorter period of time. Therefore, the higher urine output in the octreotide group during the three stages of OLT in the present study showed the hemodynamic effects of octreotide on renal and splanchnic circulation. On the other hand, no significant difference was found between the two groups regarding serum creatinine as a renal function marker because octreotide was just used during the operation time.

In our study, MAP was higher in the octreotide group compared to the control group and the two groups were similar regarding the infusion dose of norepinephrine consumption. This is due to the fact that octreotide is not a direct vasoconstrictor. Therefore, our findings were similar to those obtained by Wiest R et al. indicating that octreotide enhanced the vasoconstrictor effect of norepinephrine (22).

This study had some limitations. First, we should have followed renal function by other markers, such as Neutrophil Gelatinase-Associated Lipocalin (NGAL). Second, the dose of octreotide should be increased gradually for augmenting the systemic effect of this drug on systemic blood pressure.

Because anesthesiologists usually use vasopressin during OLT operation, further studies are recommended to compare the effects of octreotide and vasopressin on hemodynamic parameters, urine output, and postoperative renal function during OLT.

In this randomized, double-blind, placebo-controlled clinical trial, seventy nine patients who had undergone OLT were randomly allocated into two groups. In the octreotide group, the patients received octreotide infusion from the start of the operation. In the control group, on the other hand, the patients received physiologic saline infusion instead of octreotide. In the octreotide group, urine output and MAP during the three stages of OLT were significantly higher compared to the control group (P < 0.05). Moreover, no significant difference was found between the two groups regarding baseline and postoperative creatinine (P > 0.05). In conclusion, octreotide infusion could help maintaining better renal perfusion and urine output during anesthesia for OLT. In addition, octreotide infusion could augment the vasoconstrictor effect of norepinephrine and improve the patients' MAP.
